# Severe thyrotoxicosis induced by tislelizumab: a case report and literature review

**DOI:** 10.3389/fonc.2023.1190491

**Published:** 2023-10-02

**Authors:** Liman Huo, Chao Wang, Haixia Ding, Xuelian Shi, Bin Shan, Ruoying Zhou, Ping Liang, Juan Hou

**Affiliations:** ^1^ Department of Pharmacy, Fourth Hospital of Hebei Medical University, Shijiazhuang, China; ^2^ Department of Hepatobiliary Surgery, Fourth Hospital of Hebei Medical University, Shijiazhuang, China; ^3^ Department of Endocrinology, Fourth Hospital of Hebei Medical University, Shijiazhuang, China; ^4^ Department of Pain, Fourth Hospital of Hebei Medical University, Shijiazhuang, China; ^5^ Department of Pharmacy, Anguo Hospital, Baoding, China

**Keywords:** tislelizumab, immune-related adverse events, hyperthyroidism, hypothyroidism, thyroiditis

## Abstract

Immune checkpoint inhibitors (ICIs) have made significant breakthroughs in the treatment of a variety of malignancies. As its use increases, the unique immune-mediated toxicity profile of ICls are becoming apparent. We report a case of immune-related endocrine adverse events (irAE) in a patient with hepatocellular carcinoma treated with anti-programmed cell death protein 1 (PD-1) (tislelizumab). Although many irAEs have been reported, few cases of severe thyrotoxicosis have been described after immunotherapy in the literature. We present the case of a 49-year-old male who experienced a Grade 3 tislelizumab-related adverse reaction according to Common Terminology Criteria for Adverse Events (CTCAE5.0) and received methylprednisolone, thiamazole, and levothyroxine sodium tablets. Early identification of irAEs, risk factors, regular monitoring, use of steroids and/or immunoglobulins, and adjuvant supportive care are critical to the clinical prognosis of patients. It should be underlined that the tumor benefits of ICI therapy outweigh the risks associated with ICI-induced endocrine disorders, and ICI treatment should not be stopped or delayed except in rare cases (adrenal crisis, severe thyrotoxicosis). The familiarity of healthcare professionals with irAEs of the thyroid when thyrotoxicosis occurs is important to facilitate an effective diagnosis and appropriate treatment of this increasingly common thyroid disorder.

## Introduction

Immunotherapy has made a significant breakthrough and has evolved to be a standard treatment regimen in cancer treatment since the 1990s (1). In recent years, ICIs have emerged as a powerful class of immunotherapeutic medicine and are now approved for advanced malignancies by the US Food and Drug Administration. Monoclonal antibodies targeting ICIs mainly block negative regulators of T cell activation by targeting cytotoxic T lymphocyte-associated antigen 4 (CTLA-4): ipilimumab, programmed cell death 1 (PD-1) (nivolumab, pembrolizumab, cemiplimab), and its programmed cell death ligand 1 (PD-L1) (durvalumab, atezolizumab, avelumab) (2–4). PD-1 is a negative regulator of T cell activity and, when it interacts with its two ligands PD-L1 and PD-L2, it can limit T cell activation at various stages of the immune response. PD-1 plays a key role in tumor evasion of host immunity (5). Given their mechanism of action, it understandable that ICIs can also trigger autoimmune side effects or immune-related adverse events (irAEs) in recipients (6). These irAEs can affect multiple systems and sites, including the gastrointestinal tract, endocrine, skin, and liver (7). Among them, thyroid dysfunction (TD) is the most common endocrine irAEs, and mainly includes hyperthyroidism, hypothyroidism, and thyroiditis.

Tislelizumab is a new humanized IgG4 PD-1 inhibitor that was approved by the National Medical Products Administration (NMPA) in China for the treatment of classical relapsed or refractory Hodgkin lymphoma after at least second-line systemic chemotherapy, locally advanced or metastatic urothelial cancer, non-small cell lung cancer, and hepatocellular carcinoma in December 2019. Several clinical trials involving multiple indications for tislelizumab are ongoing (**Table 1**).

**Table 1:** Key ongoing clinical trials involving tislelizumab.

Trial name	Indication	Phase	Status	NCT
Surufatinib in combination with tislelizumab in subjects with advanced solid tumors	Metastatic solid tumor	I/II	Recruiting	NCT04579757
Safety and efficacy study of tislelizumab in combination with BCG in HR-NMIBC patients (TACBIN-01)	Urinary bladder neoplasms	I/II	Recruiting	NCT04922047
A study to evaluate the safety and efficacy of oral APL-1202 in combination with tislelizumab compared to tislelizumab alone as neoadjuvant therapy in patients with muscle invasive bladder cancer	Muscle invasive bladder cancer	I/II	Recruiting	NCT04813107
Chemoradiation plus tislelizumab for conversion therapy of locally nonresectable ESCC	Esophageal squamous cell carcinoma	I/II	Recruiting	NCT05394415
Study of tislelizumab in combination with Oxaliplatin and Tegafur for the treatment of gastric cancer with liver metastases	Liver metastases	II/III	Recruiting	NCT05325528
Tislelizumab in combination with lenalidomide in refractory and relapsed older adult patients with non-GCB DLBCL	Non-GCB/ABC diffuse large B-cell lymphoma	I/II	Recruiting	NCT04796857
Phase I/IIa study of BR790 in combination with tislelizumab in adult subjects with advanced solid tumors	Advanced solid tumor	I/II	Recruiting	NCT05505877
Tislelizumab combined with mitoxantrone hydrochloride liposome in extranodal natural killer/T cell lymphoma	Extranodal natural killer T cell lymphoma	I/II	Recruiting	NCT05464433
GEMOX combined with donafenib and tislelizumab in biliary tract cancer	Biliary tract carcinoma	I/II	Recruiting	NCT04979663
Adjuvant PD-1 antibody in combination with capecitabine for patients with ICC at high-risk of postoperative recurrence	Cholangiocarcinoma, intrahepatic	I/II	Recruiting	NCT04782804

The main side effects related to treatment are irAEs, including rash, pruritus, thyroiditis, diarrhea, hepatitis, and pneumonitis. Grade 3 and higher adverse effects caused by tislelizumab include severe skin reactions, anemia, pneumonitis, hypertension, and adrenocortical insufficiency (8–10). However, cases of autoimmune Grade 3 and higher hypothyroidism induced by immunotherapy are rare and have been poorly described. We describe a case of severe hypothyroidism induced by tislelizumab.

## Case presentation

**Figure 1:**
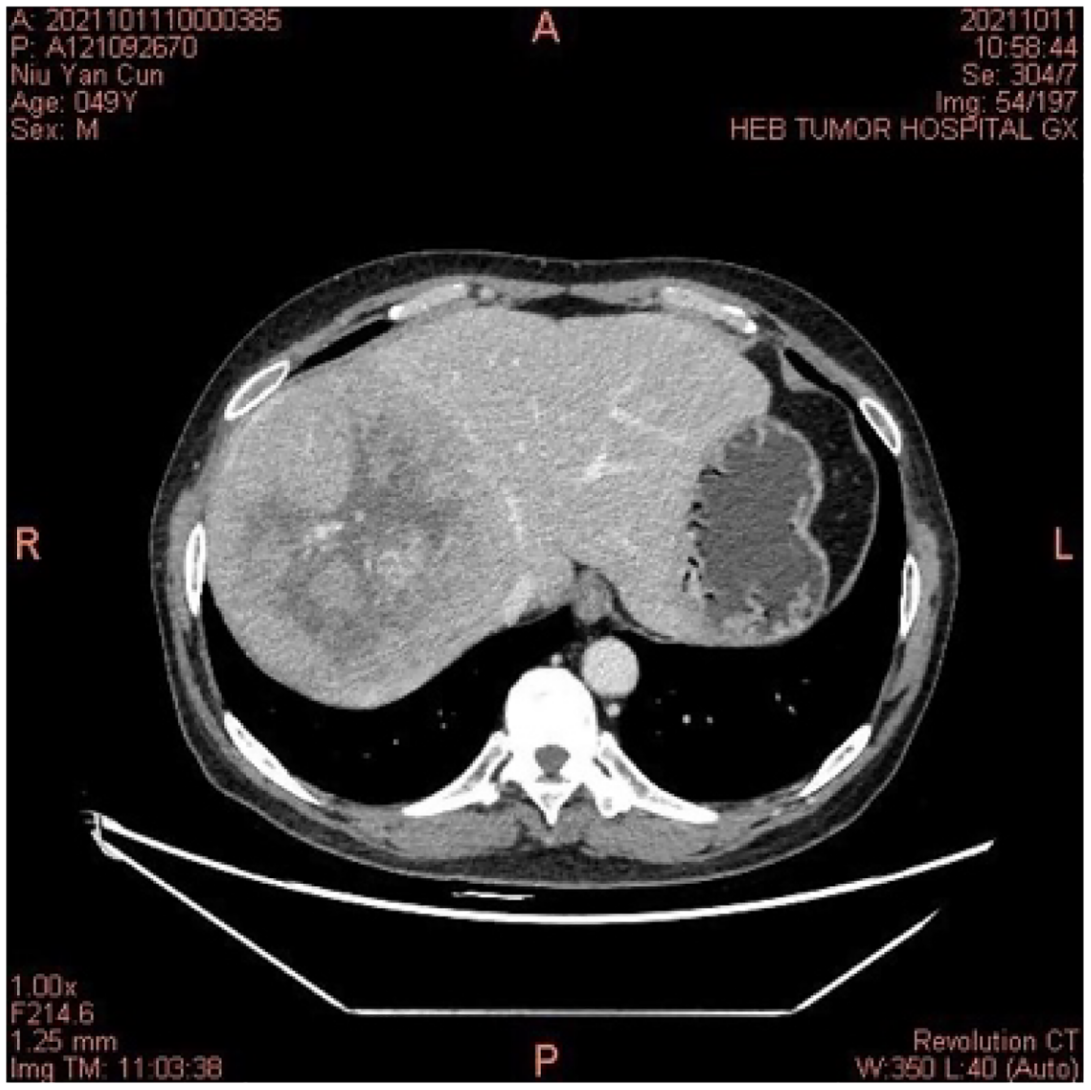
In October 2021, the computed tomography of the abdomen indicated a space-occupying lesion in the right lobe of the liver, and liver cancer was considered.

A middle-aged 49-year-old male underwent a routine physical examination in a local hospital 5 days before admission. Abdominal color ultrasound showed a solid intrahepatic mass (9 October, 2021). Laboratory results revealed alpha-fetoprotein 1.56 ng/mL, carcinoembryonic antigen 2.56 ng/mL, alanine aminotransferase 35 U/L, aspartate aminotransferase 147 U/L, direct bilirubin 34 μmol/L, and indirect bilirubin 11 μmol/L. Abdominal computed tomography (CT) revealed space-occupying lesions that involved the right lobe of the liver, with a small amount of bleeding. A subsequent upper abdominal plain scan and enhanced CT performed in our hospital (11 October) confirmed space-occupying lesions in the right lobe of the liver, about 13.5×13.9 cm in size, suggestive of liver cancer **(** **Figure 1** **)**. Laboratory tests showed hepatitis B virus surface antigen(+) and hepatitis B virus core antibody(+). During this period, the patient had no abdominal pain, abdominal distension, nausea, vomiting, or other discomfort. He had a history of hypertension of more than 3 years, regular oral administration of perindopril tert-butylamine and bisoprolol fumarate, and a history of coronary heart disease of 3 years. He underwent a coronary stent implantation at another institution 3 years prior and is currently taking aspirin, ezetimibe tablets, and rosuvastatin regularly. He had no prior history of infectious diseases or other surgical history. With regard to family history, the patient’s mother died due to esophageal cancer. The father was alive and denied any history of hepatitis-related or genetic diseases in the family. Diagnosis at admission included (i) primary liver cancer; (ii) posthepatitic cirrhosis; (iii) chronic active viral hepatitis B; (iv) coronary heart disease, coronary stent immediately after the postoperative period; and (iv) very high risk of Grade 1 hypertension. The electrocardiogram on admission showed a ventricular rate of 66 bpm, sinus rhythm, QA pattern in V1 and V2, and Q waves in III and aVF. Imaging studies revealed a huge mass, approximately 13 cm in size. Potential for surgical examination was evaluated based the patient’s physical condition and residual liver volume. Considering that the patient’s liver cancer lesion was located in the right liver and the volume was huge, it was considered that if surgical treatment was performed, the residual liver volume would be less than 40%. Thus, the risk of surgical treatment was high, and the patient was at increased risk of postoperative liver failure. Hepatic arterial infusion chemotherapy (HAIC) combined with tislelizumab was recommended. Baseline thyroid function tests included free triiodothyronine (FT3) 3.15 pmol/L (3.1–6.8), free thyroxine (FT4) 11.09 pmol/L (11–20), serum thyroid stimulating hormone (TSH) 4.25 uIU/mL (0.270–4.200), thyroglobulin antibody (TgAb) 307.1 IU/mL (0-115), and thyroid peroxidase antibody (TPOAB) 7.98 IU/mL (0–34). Two cycles of HAIC (oxaliplatin 100 mg + fluorouracil 3 g + levofolinate calcium 350 mg) combined with immunotherapy with tislelizumab 200 mg were administered on 18 October and 16 November, respectively. Thyroid function tests did not show any abnormalities during this period.

**Figure 2:**
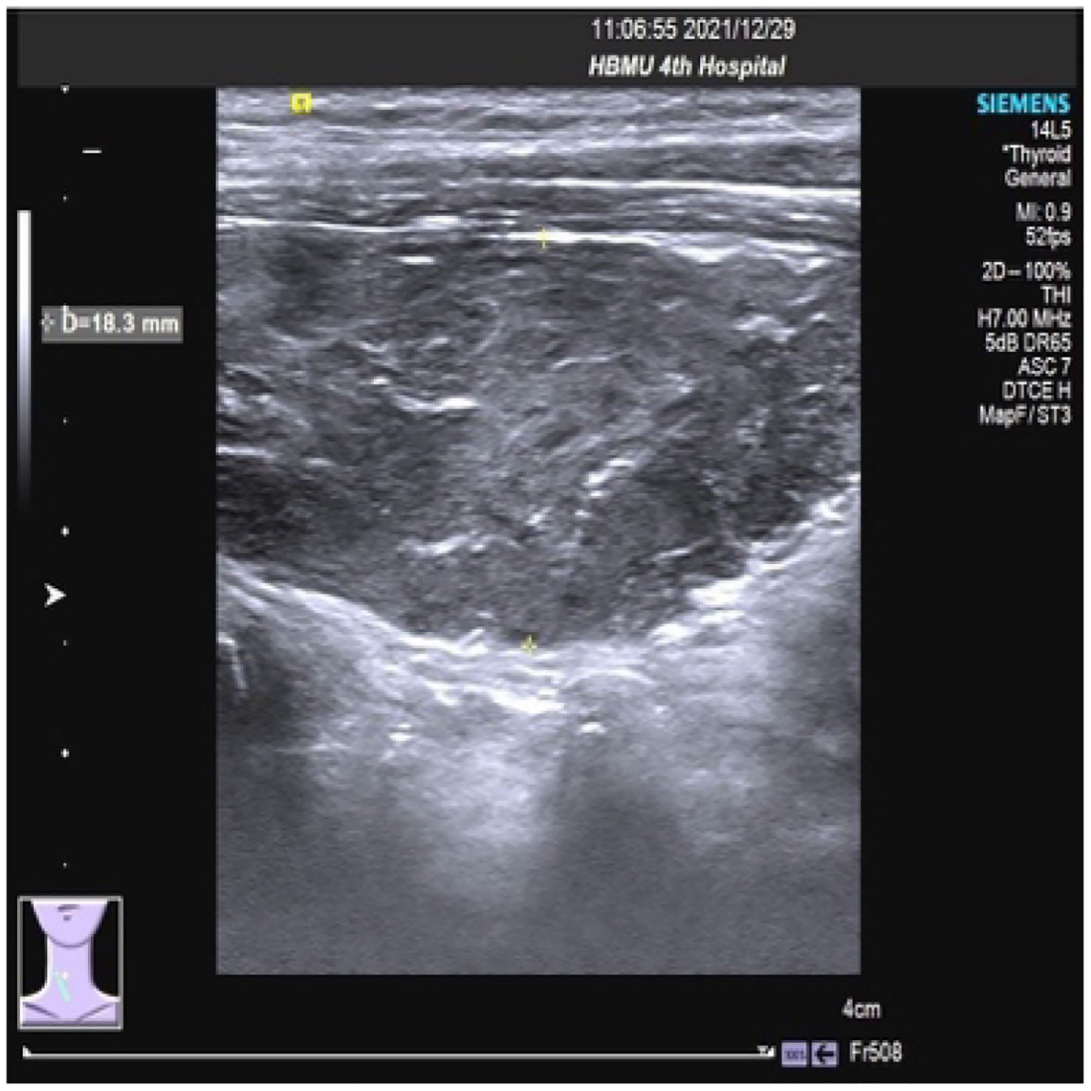
Thyroid ultrasound results showing diffuse thyroid lesions.

**Figure 3:**
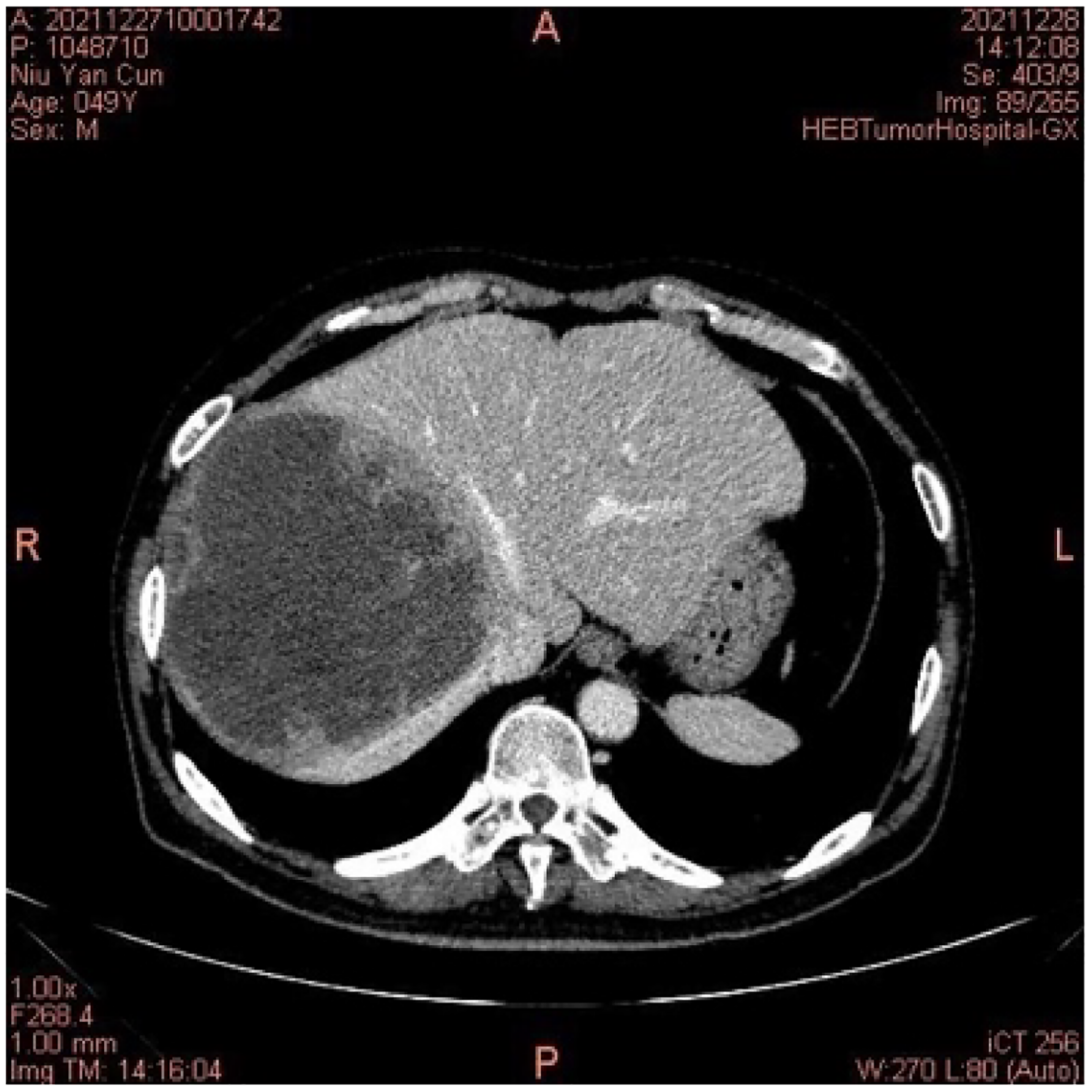
On 28 December 2021, the computed tomography of the abdomen indicated right lobe liver cancer.

On 27 December 2021, the patient experienced systemic pruritus, poor appetite with trembling of the hands, irritability, and weight loss, and severe difficulty falling asleep. Physical examination did not show yellowing of the skin mucosa or sclera of the whole body, multiple scratches on the upper limbs of the trunk and skin damage, mild bulging of the eyeballs, and no maculopapule on the skin. Thyroid function tests showed FT3 20.95 pmol/L, FT4 67.17 pmol/L, serum TSH 0.01 uIU/mL, TgAb 819 IU/mL, and TPOAB 8.24 IU/mL. Thyroid ultrasound revealed a thickness of 1.8 cm in the right thyroid lobe, 0.4 cm in the isthmus, and 1.8 cm in the left lobe. The thyroid echoes were ancestral, reduced and heterogeneous, and the diagnosis indicated diffuse thyroid lesions (**Figure 2**). ECG revealed a ventricular rate of 144 bpm, atrial fibrillation with a rapid ventricular rate. On 28 December, examination of pituitary hormone level and the pituitary magnetic resonance scan did not show any abnormalities. A complementary diagnosis of hyperthyroidism was made based on these findings. After consultation with the endocrinologist and considering the severe adverse reaction of antitumor therapy, immunotherapy was interrupted and the patient was given loratadine tablets 10 mg per os/day, thiamazole tablets 10 mg per os 2/day, and methylprednisolone tablets 28 mg/day. CT imaging on 28 December 2021 showed a large mass shadow of mixed density in the right lobe of the liver measuring approximately 14.2x13.4 cm (**Figure 3**).

Thyroid function tests on 7 January 2022 revealed FT3 2.52 pmol/L, FT4 56.4 pmol/L, and serum TSH 0.006 uIU/mL. The patient had normal diet, normal sleep patterns, no special discomfort, and hyperthyroidism improved significantly. The electrocardiogram showed a ventricular rate of 61 bpm and sinus rhythm. The third cycle of HAIC combined with tislelizumab was administered on 14 January. On 12 February, thyroid function tests showed free FT3 1.67 pmol/L, free FT4 4.58 pmol/L, serum TSH 54.97 uIU/mL, TGAB 408.6 IU/mL, and TPOAB 7.51 IU/mL. Physical examination revealed no facial edema, no tenderness in the neck, and no palpable enlargement of the thyroid gland. A supplementary diagnosis was made of immune-related thyroiditis, hypothyroidism. Levothyroxine sodium tablets 12.5 μg once daily were administered orally for thyroiditis. The CT performed on 14 February 2022 showed a large mixed density shadow in the right lobe of the liver that measures approximately 12.7x12.1 cm. Considering that the lesion was not significantly smaller than before, despite the general improvement, stable disease (SD) was evaluated for 4 months.

**Figure 4:**
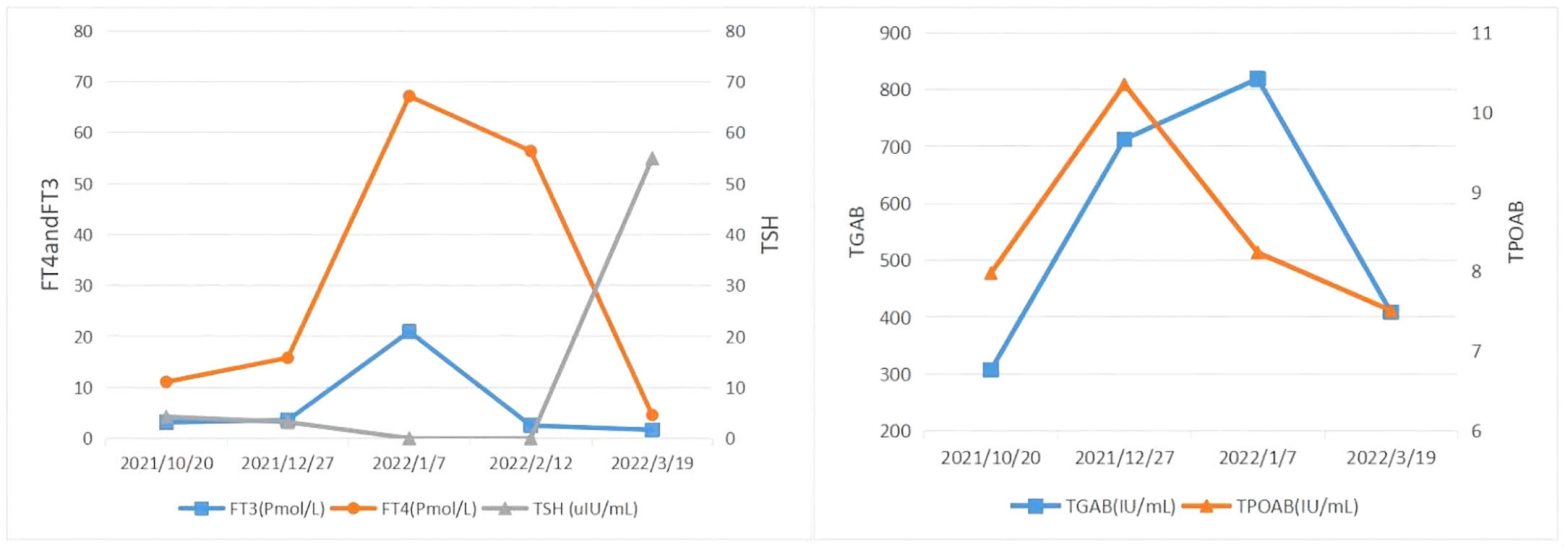
The clinical time course of the development of thyroid disease in the present patient is shown according to several clinical parameters. FT3, FT4, TSH, TgAb, and TPOAb level.

**Figure 5:**
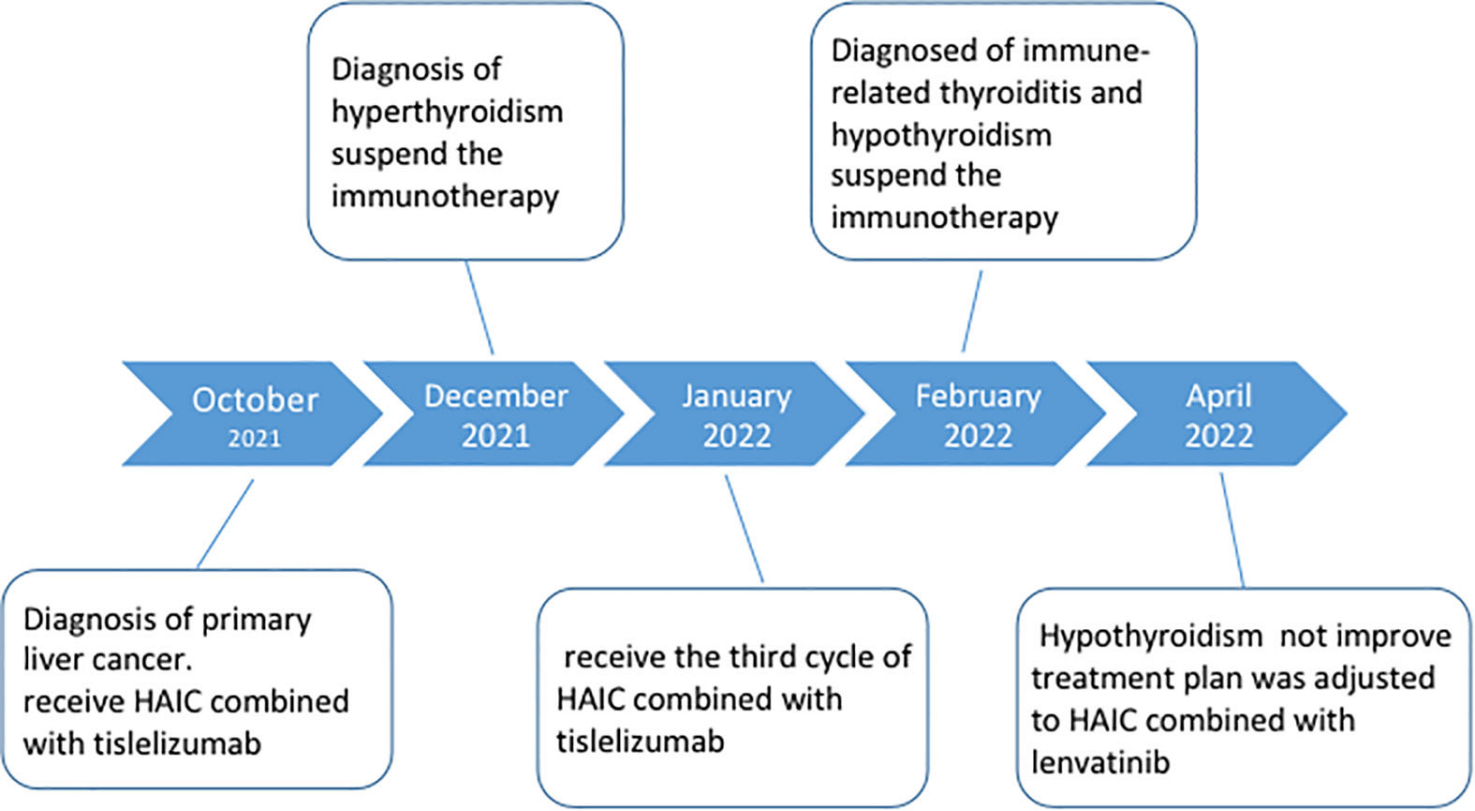
Timeline for diagnosis, treatment and immune-related adverse events.

Subsequent treatment was changed to transcatheter arterial chemoembolization (TACE) combined with lenvatinib mesylate. Three cycles of TACE combined with lenvatinib mesylate were performed from 17 February 2022 to 29 April 2022, and then treatment was changed to 8 mg of lenvatinib mesylate once daily as maintenance therapy. On 27 April 2022, reexamination of thyroid function showed free FT3 2.48 pmol/L, free FT4 5.26 pmol/L, serum TSH 62.47 uIU/mL, TGAB 575.6 IU/mL, and TPOAB 8.69 IU/mL. The clinical time course of the development of thyroid disease in the present patient is shown based on several clinical parameters (FT3, FT4, TSH, TgAb, and TPOAb levels) in **Table 2** and **Figure 4** **)**.The patient had normal diet and physical strength, increased sleep, unresponsiveness, or other discomfort. Physical examination showed no facial edema, no tenderness in the neck, and no palpable enlargement of the thyroid gland. The dose of levothyroxine sodium tablets was adjusted to 100 μg orally once daily. Follow-up examinations on 28 April 2022, 23 July 2022, and 26 August 2022, showed a large mass in the right lobe, with no significant volume changes and no significant enhancement of injury. The complete tumor evaluation was SD for 6 months, and oral lenvatinib mesylate is being continued to date. A timeline describing diagnosis, treatment, and irAEs is shown in **Figure 5**.

**Table 2:** Clinical time course of the development of thyroid disease in the present patient is shown according to several clinical parameters.

Date	FT4 (pmol/L)	FT3 (pmol/L)	TSH (mU/mL)	TgAb (IU/mL)	TPOAB (IU/mL)
2021/10/20	11.09	3.15	4.25	307.1	7.98
2021/12/27	67.17	20.95	0.01	819	8.24
2022/1/7	56.4	2.52	0.006	556.8	–
2022/2/12	4.58	1.67	54.97	408.6	7.51
2022/3/19	5.26	2.48	62.47	575.6	8.69

-, no report.

## Discussion and conclusion

Tislelizumab binds to human PD-1 with high specificity and affinity (dissociation constant, Kd 0.15 nmol/L) (21) using key epitopes GLN75, THR76, ASP77, and ARG86 present on the PD-1 receptor (11). This contrasts with nivolumab and pembrolizumab which do not require binding of these epitopes. Tislelizumab dissociates more slowly from the PD-1 receptor than nivolumab (50-fold slower) and pembrolizumab (100-fold slower). A population pharmacokinetics (popPK) model for tislelizumab showed linearity over the dose range 0.5–10 mg/kg after a single i.v. dose (12). Following a single infusion of tislelizumab 200 mg, the volume of distribution was 4.41 L and at steady state the volume was 5.247 L. Tislelizumab showed a clearance of 0.247 L/day and a half-life of 13.3 days, whereas repeated dosing in a population pharmacokinetic analysis, the clearance was 0.171 L/day and the half-life was 26 days.

Immunotherapy with ICIs uses the body’s own immune system to attack cancer cells but causes unwanted autoimmune side effects in up to 60% of patients. These irAEs may lead to treatment interruption, permanent organ dysfunction, hospitalization, and premature death. Thyroiditis is one of the most common irAEs. Studies have confirmed that thyroiditis caused by ICIs is composed of T-cell-predominant but varied immune infiltrates, mainly including γδT17 cells, CD41 Th17, and CD81 Tc17 cells. The γδT17 cells are a major subset of interleukin (IL)-17A1-producing cells and early expansion may contribute to the activation and recruitment of Th17, CD81, and other T cell populations. Following treatment with ICIs, multilineage IL-17T cells expand in the thyroid tissue, and IL-17-producing innate-like γδT17 cells increase in the thyroid gland, and adaptive Th17 cells also increase significantly in the thyroid gland. Targeting Th17 and γδT17 cell function via the IL-17A axis may therefore be a generalizable strategy for addressing type 3 immune-mediated irAEs in the future. Several hypotheses for thyroiditis include genetic susceptibility associated with the human leukocyte antigen (HLA) haplotype associated with autoimmune thyroid disease, CTLA-4 or PD-1 polymorphisms, underlying autoimmune susceptibility, disinhibited regulatory T cell function, and/or cytokine (IL-2, interferon-α)-mediated thyroiditis (13, 14).

Endocrine dysfunction is one of the most common irAEs reported in ICI clinical trials and includes hypothyroidism, hyperthyroidism, hypophysitis, primary adrenal insufficiency (PAI), and insulin-deficient diabetes mellitus (IDD) (15, 16). ICI-induced thyroid dysfunction often occurs within weeks to months of medication, with a median onset time of 18 to 123 days, and has been reported as early as 7 days and as late as 3 years, and the median onset time is shorter in patients with hyperthyroidism than in hypothyroidism, and 80% of patients with hyperthyroidism subsequently progress to hypothyroidism, with a median time to develop this evolution of 4 to 7 weeks (17, 18).

The incidence of ICI-related thyroid dysfunction depends on two factors, one is the type of ICI and the other is the form of treatment (monotherapy or immune combination therapy). It has been reported that CTLA-4 inhibitors result in ICI-related thyroid dysfunction in 1% to 5%, and in 5% to 10% following PD-1 or PD-L1 inhibitor treatment, whereas the incidence of thyroid dysfunction is as high as 10% to 20% during immune combination therapy (19, 20). The incidences of hypothyroidism with monotherapy and with different types of ICI drug immunization combinations were further analyzed in two meta-analyses on the prevalence of endocrine disorders associated with ICIs published by de Filette et al. (20) and by Barroso-Sousa et al. (19).

The reported incidence of tislelizumab related thyroid dysfunction has varied widely across different clinical trials involved lung cancer, esophageal cancer, gastric/esophageal junction cancer, urothelial cancer, and classical Hodgkin’s lymphoma (22–28). In the clinical trials, the incidence of hypothyroidism was 6% to 20%, the incidence of hyperthyroidism was 2.7% to 6%, and all were of Grade 1 to 2.

In our data set, there was a rare incidence of Grade 3 hypothyroidism and of hyperthyroidism events (0.2%). A retrospective analysis of the US Food and Drug Administration adverse event reporting system reported that 9.3% (171/1842) of patients presented with hyperthyroidism before hypothyroidism (29). Lee et al. found that 80% (28/35) of 35 patients with ICI-associated thyrotoxicosis eventually developed hypothyroidism (30). In addition, marked thyrotoxicosis was associated with prolonged progression-free survival (PFS) (HR 0.68, 95% CI 0.49 to 0.94; *P* = 0.02) and overall survival (OS) (HR 0.57, 95% CI 0.39 to 0.84; *P* = 0.005); however, there was no association between hypothyroidism and cancer outcomes (31). A systematic review of 47 studies showed that thyroid irAEs occurring during ICI therapy were associated with improved OS and PFS, and ICI-induced antitumor immunity and autoimmunity were associated, particularly in the setting of significant thyroid dysfunction. Other endocrine irAEs, such as hypophysitis and diabetes mellitus, are very rare, and treatment methods or disease processes themselves may decrease survival (32–34).

Potential risk factors for thyroid dysfunction caused by ICIs include the presence of antithyroid antibodies at baseline, higher TSH values at baseline, and dose, duration, sex, and higher BMI values in patients receiving anti-PD-1 antibody therapy, which may be associated with a high-risk of thyroid dysfunction (31, 35). In addition, thyroid autoantibodies may play a role in thyroid dysfunction associated with ICIs (36). Maekura et al. described 5 patients who presented ICI-associated hypothyroidism who were positive for at least 1 thyroid autoantibody, and of these, 4 (80%) were positive for both TgAb and TPOAb at baseline (37). A retrospective study by Iwama et al. reported patients with positive baseline TgAb or TPOAb before immunotherapy had a higher incidence of thyroid dysfunction than patients with negative baseline TgAb or TPOAb levels (38). A prospective cohort study demonstrated that patients who were positive for TgAb or TPOAb were more likely to develop thyroid dysfunction after PD-1 inhibitor therapy (39). TgAb and TPOAb are risk factors for thyroid dysfunction induced by ICIs. The mechanism may be related to the fact that ICIs inhibit PD-1 signaling in follicular helper T cells, promote follicular helper T cell proliferation, increase TgAb and TPOAb levels, induce thyroid immune function loss, and thus influence thyroid function. Pollack et al. reported an increased risk of thyroid dysfunction in patients treated with PD-1 inhibitor with higher baseline TSH levels (*P* = 0.05) and a significantly higher risk of TD in patients with baseline TSH levels >2.19 mIU/mL (OR = 3.46, 95% CI 1.2-9.8) (40).

Another clinical study reported that thyrotrophin receptor antibodies (TRAbs) tested in 6 of 7 cases of irAE-associated TD, were negative, which makes Graves’ disease caused by thyroid autoantibody TRAbs unlikely to be the main cause of irAEs-associated TD (41).

Multivariate analysis showed that disease duration (≥1a), thyroid color ultrasound revealing no nodules, and female sex were independent risk factors for the development of thyroid dysfunction, whereas patients with characteristics of female sex, disease duration (≥1 a), thyroid color ultrasound revealing no nodules, and elevated BMI were more likely to develop thyroid dysfunction (42–44). Drug dose may also be another important risk factor for thyroid injury caused by ICIs. The onset of irAEs related TD appears to be closely related to the dose administered and usually occurs after 2 to 4 infusions (45). Thus, baseline screening for thyroid function before initiating ICIs is particularly important, and patients with high thyroid antibodies before initial treatment are likely to develop thyroid dysfunction.

Our patient presented with hyperthyroidism during treatment cycle 3 (Day 65) of HAIC combined with tislelizumab for advanced hepatocellular carcinoma, which progressed to hypothyroidism during Cycle 5. Tislelizumab is a PD-1 inhibitor that predisposes to thyroid dysfunction. At present, there has been no relevant report describing thyroid dysfunction associated with HAIC treatment. In addition, our patient had no previous history of underlying thyroid disease, only thyroglobulin antibody was high before treatment, and other thyroid examination indicators were normal. Therefore, the patient may have developed thyroid toxicity caused by tislelizumab. The patient was assessed as having Grade 3 thyrotoxicosis and Grade 1 hypothyroidism according to the CTCAE 5.0 evaluation criteria (46).

In terms of immune-related hyperthyroidism, Chinese Society of Clinical Oncology/National Comprehensive Cancer Network (CSCO/NCCN) guidelines indicate that ICIs can be continued in patients exhibiting hyperthyroidism of Grade s 1 to 4 and β-blockers may treat symptoms, while further assessment of Graves’ disease is required if persistent thyrotoxicosis occurs (47, 48). ESMO recommends that thyrotoxicosis be treated symptomatically with propranolol or atenolol, and carbimazole should be considered if thyrotropin receptor antibody is positive. Prednisolone 0.5 mg/kg should be considered for thyroiditis with pain and gradually discontinued, and if it does not improve, ICIs should be discontinued and reconsidered after symptoms are controlled (49). The ESE clinical practice guidelines do not recommend antithyroid drug therapy (such as thiamazole or radioiodine) for most cases of hyperthyroidism unless thyrotoxicosis persists for 6–8 weeks or the patient has features of Graves’ disease (i.e., ocular findings, thyroid enlargement) or is positive for thyrotropin receptor antibody (50), because thyrotoxicosis is not caused by excessive thyroid hormone synthesis, but by thyroid destruction (51). Our patient discontinued treatment with the ICI tislelizumab, and in accordance with the package insert for Grade 2 or 3 hyperthyroidism, ICIs were suspended until the adverse reaction recovered to Grade 0-1, and anti-thyroid drugs were administered as needed; for Grade 4 hyperthyroidism, the drug was permanently discontinued. It has been shown that over a 16-month period, of 13/90 and 3/13 patients receiving anti-PD1 monotherapy and or combination with ipilimumab, respectively, the hyperthyroid phase ended spontaneously in all 12 patients with thyrotoxicosis without any pharmacological intervention with antithyroid drugs, but all 16 patients with thyroid dysfunction eventually required long-term levothyroxine replacement. However, thyroid dysfunction did result in ICI treatment interruption, with the longest duration of interruption being 20 weeks (mean 2; range 0–20). Following treatment for thyroid dysfunction, all patients continued ICI immunotherapy (52). Of 657 patients treated with ICI, 43 (6.5%) developed thyrotoxicosis. During the thyrotoxicosis phase, 14 (33%) patients presented with symptoms, palpitations being the most common symptom, followed by tremor, fear of heat, weight loss, and fatigue, atrial fibrillation with rapid ventricular rate, ICI therapy was not interrupted, and conservative treatment with β-blockers was sufficient for all symptomatic patients (53). Because our patient presented with Grade 3 hyperthyroidism, immunotherapy was discontinued and thiamazole tablets 10 mg b.i.d. and methylprednisolone Tablets 28 mg q.i.d., were administered concomitantly. In accordance with the guidelines and relevant literature, since our patient developed atrial fibrillation with a rapid ventricular rate, it was recommended to that β-blockers be considered to relieve symptoms without any administering antithyroid drugs (such as thiamazole tablets), unless thyrotoxicosis lasted for 6 to 8 weeks or the patient presented features of Graves’ disease (i.e., ocular manifestations, thyromegaly); thus, in such cases therapy with thiamazole tablets presents limitations.

The clinical presentation and impact of IrAE are diverse and complex, and the management of patients using ICI often requires a balance between efficacy, toxicity, and specific treatments and active multidisciplinary collaboration. Patients with mild to moderate irAEs had longer OS compared to patients without irAEs. That is, the occurrence of moderate to low grade immune-related adverse events is associated with improved survival. High-grade irAEs, on the other hand, can be life-threatening and may require discontinuation of therapy or suppression of systemic immunity, which may counteract the effects of ICIs (54). Therefore, severe thyrotoxicity caused by tislelizumab treatment may have some impact on patients with hepatocellular carcinoma. Data suggests that patients with irAEs who do not respond to treatment before onset may benefit from retreatment, and that patients who respond objectively before irAEs have similar progression-free survival and overall survival in the retreatment and off-treatment arms, and that approximately 25-50% of patients relapse after retreatment with anti-PD-1/PD-L1 antibodies. This patient had an objective response before tislelizumab caused irAEs, and suspension of treatment may have little effect on progression-free survival and overall survival, but grade 3 irAEs may be life-threatening. Therefore, to minimize the incidence of irAEs, full consideration should be given to patient tolerability and the severity of irAEs.

Immune-related hypothyroidism is usually permanent and requires thyroid hormone replacement therapy (55). The CSCO/NCCN/ESMO guidelines recommendations are very similar: clinical observation is only required when Grade 1 hypothyroidism is asymptomatic and no treatment is required, ICIs are continued, and levothyroxine sodium tablets are used for replacement therapy when Grade 2-4 hypothyroidism continues (49, 56, 57). The CSCO guidelines emphasize that hypothyroidism and other endocrine toxicities (such as diabetes) do not require glucocorticoid therapy, but alternative hormone therapy is recommended. ICIs may be continued if only cutaneous or endocrine symptoms are present. The NCCN recommends about 1.6 μg/kg daily oral levothyroxine (dose reduction in older patients or in patients with cardiac disease), and the ESMO guidelines recommend smaller doses (0.5 to 1.5 μg/kg) for thyroid hormone supplementation. In the package insert of tislelizumab, for Grade 2 or 3 hypothyroidism, ICIs should be suspended until the adverse reaction recovers to Grade 0-1, and thyroid hormone replacement therapy should be started as needed; if acute thyroid inflammation is suspected, discontinuation of tislelizumab and corticosteroid therapy may be considered. For Grade 4 hypothyroidism, treatment with tislelizumab must be permanently discontinued. Thyroid function should be monitored to ensure appropriate hormone replacement therapy. In one study of 657 patients treated with ICI, 37 (84%) developed hypothyroidism and subsequently started thyroid hormone replacement therapy. Four (9%) patients recovered from transient hypothyroidism and did not require levothyroxine, and 2 patients died before developing hypothyroidism. The median levothyroxine dose required to achieve euthyroid status was 1.2 μg/kg (range 0.25–3 μg/kg). Patients in this study were followed for a prolonged period (>14 months) after the onset of hypothyroidism, and all patients who started levothyroxine remained on thyroid hormone replacement therapy at the last follow-up, suggesting that hypothyroidism may require lifelong treatment (53). In this patient, tislelizumab 200 mg combined with HAIC was continued for 1 cycle after significant improvement of hyperthyroidism, and the patient progressed to hypothyroidism (TSH 54.97 μIU/mL) and was supplemented with levothyroxine sodium tablets 12.5 μg/day. Thyroid function was followed up 2 months later: FT3 2.48 pmol/L, FT4 5.26 pmol/L, TSH 62.47 μIU/mL. Levothyroxine sodium tablets were dose adjusted to 100 μg q.i.d. According to the CTCAE 5.0 evaluation criteria, for treatment of hypothyroidism Grade 1, the patient was asymptomatic, and it was reasonable to continue the use of tislelizumab. Our patient had coronary heart disease and hypertension; thus, in accordance with the guidelines and the package insert of levothyroxine sodium tablets, special attention was paid at the start of thyroid hormone therapy, and a lower initial dose was selected. Therefore, it was considered advantageous to carefully use levothyroxine sodium tablets in this patient.

The patient’s perspective was as follows: after 2 cycles of tislelizumab, I experienced problems including poor appetite, shaking hands, atrial fibrillation, irritability, weight loss, and difficulty falling asleep. The clinician paid great attention to me throughout the treatment and found that it may be that I had had a significant adverse repose response to tislelizumab, which had improved after providing symptomatic treatment. At present, I am pleased to know that the cause of the problem was resolved by oral treatment with levothyroxine sodium tablets. I would like to thank the clinician for making this diagnosis, which improves my perception of anti-cancer therapy.

In conclusion, we report for the first time a case of severe thyroiditis caused by tislelizumab and discuss the treatment of immune-related thyroiditis, which suggests that it is necessary to improve the clinical understanding of PD-1-inhibitor-induced thyroid dysfunction and strengthen multidisciplinary collaboration in the management of such patients, especially within 1 – 2 months of treatment initiation, to monitor thyroid-related parameters, and once a patient develops immune-related thyroiditis, the patient’s condition should be comprehensively assessed and appropriate treatment should be given for symptomatic treatment and treatment if necessary to improve the patient’s prognosis.

## Data availability statement

The original contributions presented in the study are included in the article/supplementary material. Further inquiries can be directed to the corresponding authors.

## Ethics statement

Written informed consent was obtained from the individual(s) for the publication of any potentially identifiable images or data included in this article. The patients/participants provided their written informed consent to participate in this study. Written informed consent was obtained from the individual(s) for the publication of any potentially identifiable images or data included in this article. Written informed consent has been obtained from the participant/patient for the publication of this case report.

## Author contributions

PL, JH: Conceptualization, project administration, funding acquisition, writing–review and editing. LH, CW: Writing – review and editing. HD, XS: Supervision. RZ, BS: Writing – original draft, formal analysis. All authors contributed to the article and approved the submitted version.
